# Mosaic pathogenic *PRKAG2* variant and pathologic diagnosis of glycogen storage disease in an infant with negative whole-genome sequencing

**DOI:** 10.1016/j.jhlto.2026.100562

**Published:** 2026-04-11

**Authors:** Vinicius Cabido, Yue Huang, Nancy Halnon, Judith Fan, Marlin Touma, Gregory A. Fishbein, Majid Husain

**Affiliations:** a Division of Pediatric Cardiology, UCLA Mattel Children's Hospital, 200 Medical Plaza, Suite 330, 90095, Los Angeles, CA; b Division of Genetics, Department of Pediatrics, University of California, La Jolla, San Diego, CA; c Department of Human Genetics, David Geffen School of Medicine at UCLA, Los Angeles, CA; d Division of Neonatology and Developmental Biology, UCLA Mattel Children's Hospital, 757 Westwood Plaza, 90095, Los Angeles, CA; e Department of Pathology, David Geffen School of Medicine at UCLA, Los Angeles, CA

**Keywords:** PRKAG2, Biventricular hypertrophy, Cardiomyopathy, Heart transplantation, Mosaicism

## Abstract

PRKAG2 syndrome is a rare, autosomal dominant, glycogen storage disease of the heart characterized by a triad of ventricular hypertrophy, progressive conduction abnormalities, and pre-excitation. In severe cases, patients may present with neonatal onset of significant ventricular dysfunction with outflow obstruction resulting in sudden cardiac death or the need for transplantation. We present a unique case of an infant with non-diagnostic whole genome sequencing who developed progressive biventricular hypertrophy, ultimately requiring mechanical circulatory support and heart transplantation. Pathological evaluation of the explanted heart revealed excessive free glycogen accumulation within cardiomyocytes. Genetic testing identified a mosaic *PRKAG2* pathogenic variant, confirming the diagnosis of PRKAG2 syndrome. This case suggests mosaicism should be considered in the diagnostic workup of early-onset cardiomyopathy.

## Background

PRKAG2 syndrome (PS) is a rare, early-onset autosomal dominant metabolic disease caused by pathogenic variants in the *PRKAG2* gene, leading to glycogen accumulation within cardiomyocytes resulting in marked hypertrophy.^1^ The estimated prevalence of PS is 0.23%-1% in patients with suspected hypertrophic cardiomyopathy.^2^ Cardiac manifestations include a triad of eccentric and/or concentric non-sarcomeric ventricular hypertrophy, progressive conduction abnormalities, and pre-excitation. Clinical spectrum ranges from asymptomatic to significant ventricular dysfunction with outflow obstruction resulting in sudden cardiac death or the need for transplantation.^3^ We present a unique case of mosaic *PRKAG2* pathogenic variant detected in explanted myocardial tissue in an infant with non-diagnostic whole genome sequencing (WGS). Informed consent for publication of this case report was obtained from the patient’s parents.

### Case report

**Figure 1:**
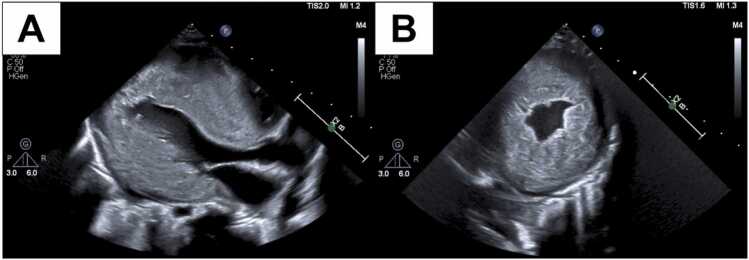
Initial transthoracic echocardiogram. (A) Parasternal long and (B) short axis views demonstrating significant concentric LVH without LVOT obstruction based on spectral Doppler mapping (not shown).

A 4-week-old infant with a postnatal diagnosis of biventricular hypertrophy and Wolff-Parkinson-white syndrome was evaluated for heart transplantation. Echocardiogram demonstrated mild right ventricular hypertrophy, severe concentric left ventricular hypertrophy (LVH) without left ventricular tract outflow obstruction, with the interventricular septum thickness in diastole 1.94 cm (Z +22.4) and the left ventricular posterior wall dimension in diastole 1.50 cm (Z +17.3) (Figure 1). Chromosomal microarray and WGS were unremarkable except for a heterozygous variant of unknown significance in the *NEXN* gene (c.1302del:p.Ile435fs(NM_144573.4)). This variant was reported to be unlikely to the cause of his clinical phenotype based on available genomic data.

The patient developed worsening biventricular hypertrophy, with obliteration of the left ventricular tract outflow and worsening ventricular function. He underwent Berlin Heart EXCOR left ventricular assist device placement, followed by veno-arterial extracorporeal membrane oxygenation for biventricular failure due to progressive right ventricular dysfunction and right ventricular outflow tract obstruction. At 5 months of age, he underwent successful heart transplantation.

**Figure 2:**
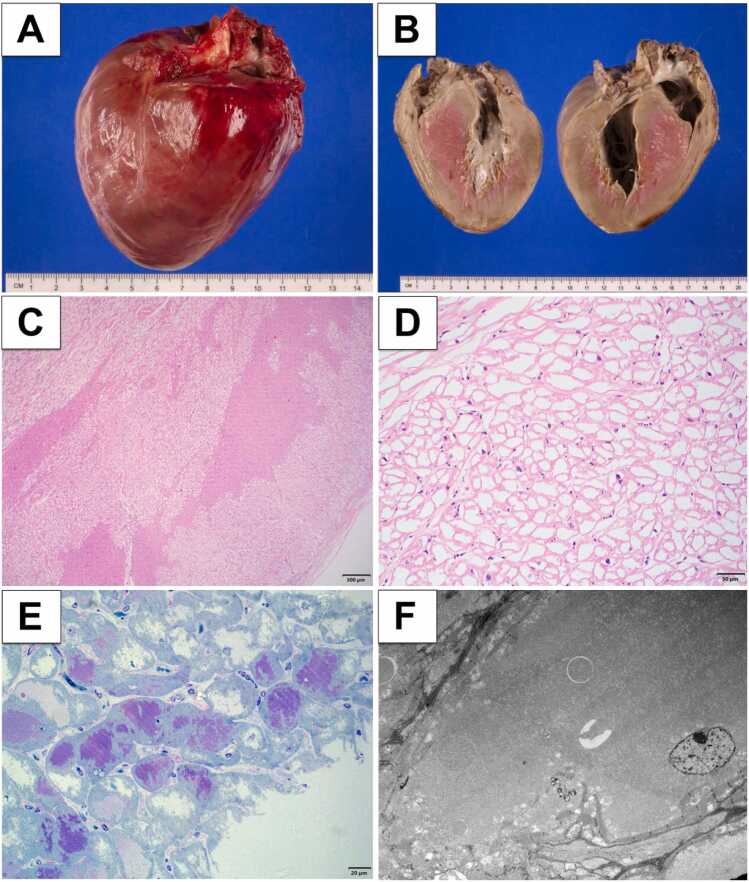
Pathology results. (A) Macroscopic examination notable for cardiomegaly. (B) Gross specimen - LV long axis cut of the heart showing massive concentric LVH. (C & D) Low and high-power H&E-stained sections showing sheets of vacuolated myocytes. (E) Toluidine-blue stained section showing glycogen (purple) within the vacuolated myocytes. (F) Transmission electron microscopy showing abundant glycogen in myocyte.

Macroscopic examination of the explanted heart revealed cardiomegaly (Figure 2A) and massive concentric LVH (Figure 2B). Ultrastructural studies of the explanted tissue revealed excess free glycogen in cardiomyocytes, consistent with glycogen storage disease (Figure 2C-F). Periodic acid-Schiff with and without diastase were negative, likely due to the “glycogen drift” phenomenon. Conventional chemical fixation and standard paraffin processing result in shifting of glycogen particles from cells because glycogen is water-soluble and easily lost during aqueous processing steps. There are several papers that acknowledge this phenomenon and attempt to correct it.^4^, ^5^, ^6^ The reason the glycogen is retained in the toluidine blue thick section is because of how the tissue is processed for transmission electron microscopy. Transmission electron microscopy processing employs glutaraldehyde fixation followed by osmium tetroxide post-fixation and embedding in epoxy resins.^7^ This protocol rapidly cross-links proteins and stabilizes water-soluble glycogen particles before extraction can occur. The osmium post-fixation further reduces membrane permeability, preventing glycogen loss.

The DNA isolated from myocardial tissue revealed a pathogenic variant in the *PRKAG2* gene (c.1592G>A;p.Arg531Gln(NM_016203.3:)) which is associated with autosomal dominant glycogen storage related to Wolff-Parkinson-white and hypertrophic cardiomyopathy - matching this patient’s phenotype. This was done using a commercial cardiomyopathy and glycogen storage disease gene panel consisting of 147 different genes (Invitae Corporation, San Francisco, CA, 94103). The panel was performed through a different laboratory than the WGS, and it identified a *NEXN* p.Ile435fs variant, a pathogenic *POLG* variant, c.752C>T; p.Thr251Ile (NM_002693.2), and 3 variants of uncertain significance: POLG, c.1760C>T (p.Pro587Leu); SLC37A4, c.492C>A; p.Ser164Arg (NM_001164277.1), and VCL, c.455A>G; p.Glu152Gly (NM_014000.2). Because the *NEXN* and *POLG* genes are associated with autosomal recessive dilated cardiomyopathies, these pathogenic variants were not suspected to be the cause of the cardiomyopathy given their heterozygous state.

## Discussion

To our knowledge, this is the first report highlighting a mosaic *PRKAG2* pathogenic variant diagnosed from explanted myocardium after non-diagnostic serum-based WGS. While the diagnosis of PS is typically made using patient serum to identify such pathogenic variants, the abundance of unique variants and significant heterogeneous nature of the disease spectrum can pose quite a diagnostic challenge.^8^ There are several established *PRKAG2* pathogenic variants with different variable penetrance and phenotypes, including some reports of intrafamilial phenotypic variability despite family members carrying the same inherited variant.^9^ From an imaging standpoint, there several findings associated with PS, but none that are diagnostically specific. Certain established echocardiographic features that may help distinguish PS from other sarcomeric cardiomyopathies, including lower baseline heart rate and global longitudinal strain, but these overlap with other types of cardiomyopathies.^10^ Some centers may use cardiac magnetic resonance for diagnostic purposes, which may demonstrate findings such as asymmetric LVH, reduced T1, and normal T2-relaxation times. Once again, these are not specific to PS, and it is important to note that most published data are from studies with small sample sizes, leading to less reproducible and generalizable image findings.^11^ We were not able to obtain a cardiac magnetic resonance for our patient since he had a left ventricular assist device in place, and given his critical clinical condition at the time, an endomyocardial biopsy was not performed.

This case emphasizes the diagnostic value of myocardial tissue testing when clinical suspicion for PS remains high despite negative blood-based genetic studies. Endomyocardial biopsy prior to transplantation or explant tissue sequencing should be considered in similar scenarios to uncover mosaic or tissue-limited pathogenic variants, guiding appropriate management and genetic counseling. In terms of future family planning, the patient’s mosaicism is believed to be a de novo phenomenon, as the WGS included parents as comparators, significantly decreasing the risk of this severe phenotype in relatives. Relatives may still be at risk for dilated cardiomyopathy given the presence of the *NEXN* variant.

## Declarations of interest

The authors declare that they have no known competing financial interests or personal relationships that could have appeared to influence the work reported in this paper.

## References

[1] Calore M. (2017). The PRKAG2 gene and hypertrophic cardiomyopathy: an energetically imbalanced relationship. Am J Physiol Heart Circulatory Physiol.

[2] Banankhah P., Fishbein G.A., Dota A., Ardehali R. (2018). Cardiac manifestations of PRKAG2 mutation. BMC Med Genet.

[3] Porto A.G., Brun F., Severini G.M. (2016). Clinical spectrum of PRKAG2 syndrome. Circ Arrhythm Electrophysiol.

[4] Saitoh Y., Terada N., Saitoh S., Ohno N., Fujii Y., Ohno S. (2010). Histochemical approach of cryobiopsy for glycogen distribution in living mouse livers under fasting and local circulation loss conditions. Histochem Cell Biol.

[5] Taksir T.V., Griffiths D., Johnson J., Ryan S., Shihabuddin L.S., Thurberg B.L. (2007). Optimized preservation of CNS morphology for the identification of glycogen in the Pompe mouse model. J Histochem Cytochem.

[6] Bendayan M., London I., Kemp B.E., Hardie G.D., Ruderman N., Prentki M. (2009). Association of AMP-activated protein kinase subunits with glycogen particles as revealed in situ by immunoelectron microscopy. J Histochem Cytochem.

[7] Mascorro J.A., Bozzola J.J. (2007). Electron Microscopy: Methods and Protocols.

[8] Lopez-Sainz A., Dominguez F., Lopes L.R., European Genetic Cardiomyopathies Initiative Investigators (2020). Clinical features and natural history of PRKAG2 variant cardiac glycogenosis. J Am Coll Cardiol.

[9] Marcu A.S., Vătăşescu R., Onciul S., Rădoi V., Jurcuţ R. (2022). Intrafamilial phenotypical variability linked to PRKAG2 mutation—family case report and review of the literature. Life.

[10] Tang L., Li X., Zhou N., Jiang Y., Pan C., Shu X. (2022). Echocardiographic characteristics of PRKAG2 syndrome: a research using three-dimensional speckle tracking echocardiography compared with sarcomeric hypertrophic cardiomyopathy. Cardiovasc Ultrasound.

[11] Pöyhönen P., Hiippala A., Ollila L. (2015). Cardiovascular magnetic resonance findings in patients with PRKAG2 gene mutations. J Cardiovasc Magn Reson.

